# Close-to-native bone repair via tissue-engineered endochondral ossification approaches

**DOI:** 10.1016/j.isci.2022.105370

**Published:** 2022-10-14

**Authors:** Sara Nadine, Inês J. Fernandes, Clara R. Correia, João F. Mano

**Affiliations:** 1 CICECO – Aveiro Institute of Materials, Department of Chemistry, University of Aveiro, Campus Universitário de Santiago, 3810-193 Aveiro, Portugal

**Keywords:** Health technology, Tissue engineering, Biomaterials

## Abstract

In order to solve the clinical challenges related to bone grafting, several tissue engineering (TE) strategies have been proposed to repair critical-sized defects. Generally, the classical TE approaches are designed to promote bone repair via intramembranous ossification. Although promising, strategies that direct the osteogenic differentiation of mesenchymal stem/stromal cells are usually characterized by a lack of functional vascular supply, often resulting in necrotic cores. A less explored alternative is engineering bone constructs through a cartilage-mediated approach, resembling the embryological process of endochondral ossification. The remodeling of an intermediary hypertrophic cartilaginous template triggers vascular invasion and bone tissue deposition. Thus, employing this knowledge can be a promising direction for the next generation of bone TE constructs. This review highlights the most recent biomimetic strategies for applying endochondral ossification in bone TE while discussing the plethora of cell types, culture conditions, and biomaterials essential to promote a successful bone regeneration process.

## Graphical abstract

**Figure:**
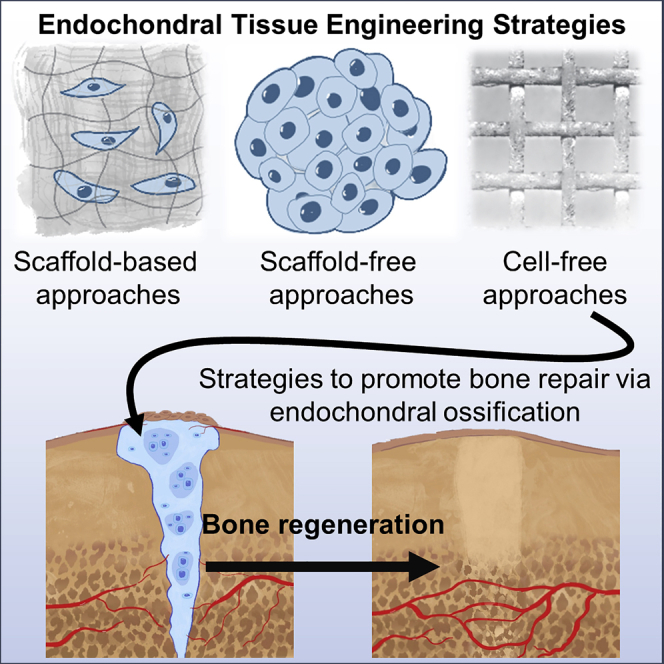

## Introduction

Bone Tissue Engineering and Regenerative Medicine (TERM) strategies aim to solve challenges related to large bone nonunions and defects caused by trauma, congenital anomalies, infection, or surgical resection. Although bone tissue presents an incredible intrinsic self-repair capability, large defects may result in the cessation of the regenerative process.^1^^,^^2^ Moreover, unhealthy conditions, such as osteoporosis, diabetes, and genetic factors, increase the chances of suffering a fracture or delayed repair.^1^ Bone grafting has been widely employed over the last years and remains the gold standard for repairing large bone defects.^2^, ^3^, ^4^ Although these surgical procedures are generally successful, 10% of the bone grafts do not fully recover, which results in bone nonunions. Furthermore, these techniques are invasive and painful and may lead to disease transmission, infection, and compatibility issues.^2^^,^^3^^,^^5^

During the last decades, less invasive surgery techniques have been emerging. For instance, kyphoplasty is used to repair spine compression fractures through a minimally invasive half-inch incision, followed by poly(methyl methacrylate) injection to create an internal cast in the fractured cavity.^6^^,^^7^ Likewise, applying robots to support or perform surgery, such as Mako, allows total hip replacement with more precision and less blood loss.^8^ However, these minimally invasive surgeries are unsuccessful in treating most large bone defects. To overcome these drawbacks, advanced bone TE strategies are being developed to replace the use of inert prostheses and improve bone grafting. Although inert biomaterials, such as bioceramics and biomedical metals, can replace the function and structure of the bone tissue, the lack of bioactivity and biodegradability makes them unattractive for long-term implantation.^9^ Biomaterial scientists aim to create smart constructs that can fully integrate with the host’s environment while improving vascularization. Therefore, the design of biomaterials has been getting closer to the architecture of the native scenario of bone.^10^

One of the main focuses of developing biomimetic engineered bone strategies is the recapitulation of the ontological processes that occur during bone formation/healing. In the native environment, bone is formed by two distinct mechanisms, namely endochondral and intramembranous ossification.^11^ Briefly, endochondral ossification (ECO) is characterized by the chondrogenic differentiation of recruited mesenchymal stem/stromal cells (MSCs), forming an initial cartilaginous template, followed by hypertrophic differentiation that will result in the release of pro-angiogenic factors. The release of such biomolecules consequently leads to the vascularization and remodeling of the previously formed cartilaginous template, giving rise to newly deposited bone tissue. In the case of intramembranous ossification (IMO), MSCs are directly stimulated to differentiate into bone cells, namely osteoblasts, which later deposit bone matrix.^12^ Most bone TE strategies have been mimicking the IMO process to generate bone tissue. Seeding undifferentiated MSCs on scaffolds, utilizing cell-free approaches loaded with bone-stimulating growth factors, adding mechanical signals, dynamic stimuli, or co-culturing with other cell phenotypes are successful examples employed to induce direct osteogenic differentiation of osteoprogenitor cells.^13^, ^14^, ^15^, ^16^, ^17^ However, one of the major barriers that hamper the translation of IMO-based TE strategies into the clinics is the lack of a functional vascular supply of the generated tissues.^18^ Some authors have observed that the implantation of MSC-seeded scaffolds in the presence of osteogenic supplementation factors affects cell viability, resulting in necrotic cores and avascular implants.^19^^,^^20^ In addition, the *in vitro* osteogenic differentiation of cell-laden scaffolds can block the entrance of the host’s regenerative macrophages, impairing the remodeling of the implanted tissue and new bone formation.^20^ The *in vitro* pre-vascularization of the IMO-engineered tissues might be a tactic to enhance the perfusion of the calcified matrix by the host’s blood vessels. More recently, the recapitulation of ECO has been increasingly explored as an alternative to overcome these limitations. One of the reasons is that cartilage cells are physiologically adapted to avascular conditions. Besides, hypertrophic chondrocytes secrete osteogenic factors to transform the cartilage template into bone tissue, whereas they also release angiogenic factors that induce tissue vascularization.^21^ Comparisons between IMO- and ECO-based strategies were already evaluated *in vitro* and *in vivo*.^22^, ^23^, ^24^ For instance, Thompson and colleagues analyzed the efficacy of ECO-based constructs over conventional IMO approaches by seeding MSCs in collagen-hyaluronic acid scaffolds and priming cells along chondrogenic or osteogenic lineages. Although both methods supported new bone formation when implanted in rat critical-sized calvarial defects, micro-CT and histomorphometry showed significantly higher levels of mature bone deposition in ECO-based constructs after 8 weeks of implantation. In addition, the histological analysis of the chondrogenically primed constructs indicated the presence of osteocytes and lamellar-like structures surrounded by osteoblasts, typical characteristics of cortical bone.^22^ Besides mature bone deposition, TE strategies based on ECO approaches were shown to influence vascularization positively compared with IMO constructs. For instance, the implantation of polycaprolactone scaffolds seeded with chondrogenic primed MSCs in mouse with critical-sized cranial defects significantly enhanced vessel infiltration compared with osteogenic primed and unprimed MSCs.^23^ Although it is also expected to observe blood vessel invasion in IMO constructs, ECO strategies have been shown to express higher levels of endothelial cell markers and pro-angiogenic factors and support greater bone remodeling.^22^^,^^25^ It is well known that during ECO, hypertrophic chondrocytes express vascular endothelial growth factor (VEGF), leading to cartilage blood vessel invasion.^26^ However, the therapeutic application of VEGF itself or loaded in scaffolds implies its release at supraphysiological levels due to its narrow therapeutic window, which may be potentially toxic.^27^ Therefore, ECO constructs are interesting from a clinical point of view, as they can continuously produce a cocktail of angiogenic molecules that may substitute the exogenous media factors usually added to scaffolds.

This review highlights the most recent and successful biomimetic strategies for applying ECO in bone TE while describing which type of cells, culture conditions, and biomaterials can be leveraged to upscale tissue-engineered hypertrophic cartilaginous grafts suitable for promoting bone regeneration through ECO. Ultimately, future trends in biomaterial science that may accelerate the ECO approach toward implementation in a clinical setting will be discussed.

## Recapitulation of the endochondral ossification process after bone fracture

**Figure 1:**
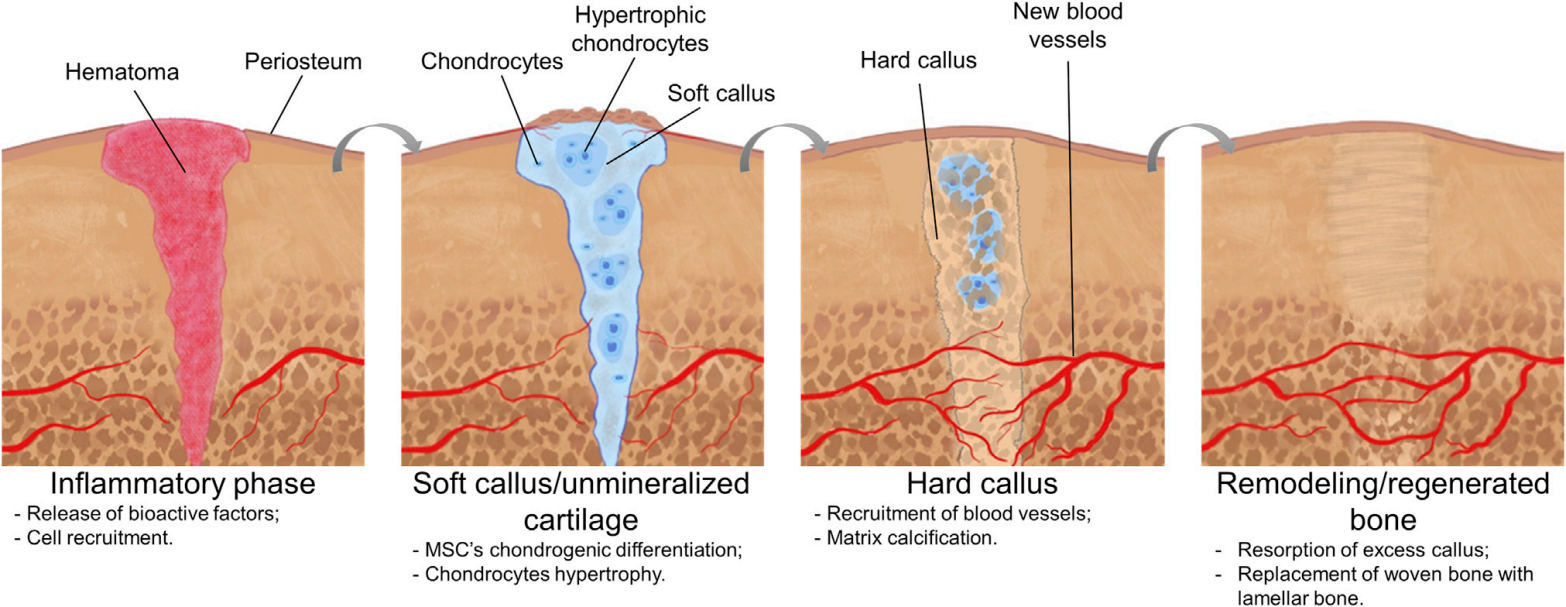
Schematic representation of the bone endochondral ossification repair process

ECO is a biological process responsible for creating every bone below the skull in human beings, except the clavicle.^2^ This process begins in the second month of gestation and goes to adulthood; however, the majority of the process occurs until birth. Most critical-sized fractures are regenerated at some level via ECO, even though IMO can co-occur. Although IMO occurs predominantly in the periosteum through the direct differentiation of MSCs into osteoblasts, ECO occurs mainly at the center of the fracture, where a cartilaginous callus is formed and remodeled into mature bone.^28^ Figure 1 represents an overview of the ECO process during fracture healing. The inflammatory phase is the first step following a fracture, and it is characterized by the release of several mediators that recruit and activate inflammatory cells. Then, molecules such as fibroblast growth factor (FGF), transforming growth factor β (TGF-β), platelet-derived growth factor (PDGF), insulin-like growth factor (IGF), and bone morphogenic proteins (BMPs) are implicated in the recruitment and differentiation of osteoprogenitor cells. After migration and differentiation of cells from the periosteum and bone marrow, endothelial cells, fibroblasts, and osteoblasts promote the granulation tissue formation at the gap zone. At the same time, macrophages regulate early angiogenesis.^29^^,^^30^ Afterward, MSCs present in the created fibrovascular tissue differentiate into chondrocytes or osteoblasts.^31^ At this stage, chondrocytes lay down extracellular matrix (ECM) rich in collagen type II (Col II), aggrecan, and sulfated glycosaminoglycans (GAGs), giving rise to cartilage callus bridges that stabilize the fracture.^32^ Inside this template, chondrocytes enter into a hypertrophic state, distinguished by the production of Col X, secreting key angiogenic growth factors, including VEGF and PDGF, to facilitate the cartilage callus vascular invasion. Then, hypertrophic chondrocytes express osteogenic markers, including alkaline phosphatase (ALP), matrix metalloproteinase (MMP)-13, osteocalcin, osteopontin, and BMP-6, to promote the calcification of the cartilage matrix.^2^^,^^18^ Following cartilage calcification, osteoblasts migrate and invade the acellular cartilage matrix along with blood vessels to produce the hard callus. More recently, it was found that osteoblasts are not just brought by the periosteal bud but also result from a chondrocyte-to-osteoblast transformation.^31^ The created marrow spaces are obtained through the apoptosis of hypertrophic chondrocytes and osteoblasts. Remodeling is the last stage of fracture repair, which is characterized by the degradation of woven bone and its replacement with mature lamellar bone. Osteoclasts are key components of callus remodeling.^33^

### Regulation of the hypertrophic stage during endochondral ossification

**Figure 2:**
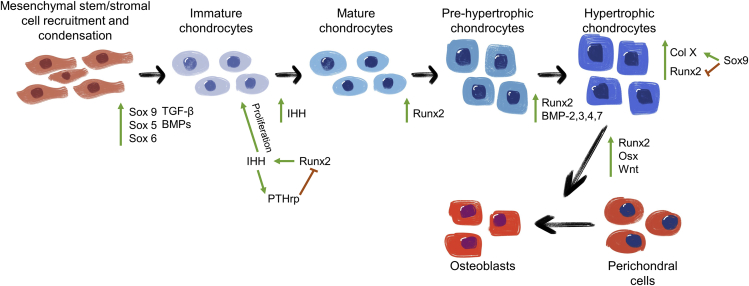
Regulation of chondrogenic differentiation and hypertrophy The transcription factor Sox9 is crucial for the chondrogenic differentiation of mesenchymal stem cells, whereas Runx2 is necessary for chondrocyte maturation.

The molecular and cellular mechanisms that control the hypertrophic stage during ECO are highly complex processes that have not been elucidated in detail. The regulation of hypertrophy is performed through local and systemic factors. Sox9, Runx2, and BMPs are transcription and growth factors crucial for the viability and development of chondrocytes, which are regulated through several signaling pathways (Figure 2).^34^ For instance, after the condensation of MSCs, the transcription factor Sox9 is expressed to promote the differentiation into chondrocytes. When Sox9 is not expressed, the cartilaginous template is not formed, as shown in a knockout mice model.^35^ After chondrogenic differentiation, the expression of Sox9 decreases because this transcription factor inhibits chondrogenic hypertrophy.^36^ The lack of Sox9 expression can also result in the absence of Col X, indicating that the expression of Sox9 is essential for the existence of a hypertrophic stage.^37^ Sox5 and Sox6 also play fundamental roles during cartilage development. Although mice deficient in Sox5 or Sox6 alone survive with mild skeletal dysplasia, the formation of cartilage in Sox5/Sox6 double-knockout mice is harshly compromised.^38^

The Indian hedgehog (IHH) factor signaling pathway promotes the chondrocytes' proliferation and differentiation, which is controlled through the IHH-parathyroid hormone-related protein (PTHrp) feedback loop. The regulation of these two pathways is made by negative feedback, which regulates the growth of the proliferative region and makes the connection between cortical and longitudinal bone formation.^39^ Briefly, PTHrp is induced by the IHH signal to promote chondrocyte proliferation, whereas PTHrp acts on PTH/PTHrp receptors (PPR) to block chondrocyte terminal differentiation. The chondrocyte hypertrophy may increase when the IHH signal is absent, and the expression of PHHrp is reduced.^40^^,^^41^ Scotti et al. highlighted the importance of the IHH pathway in the maturation of the cartilaginous template of ECO. For that, a cyclopamine derivative was used to perform a functional inhibition of the IHH pathway. Results showed that the expression of genes involved in the IHH and parathyroid hormone signaling and chondrogenic/hypertrophic and osteogenic genes were significantly reduced.^42^ Of note, IHH is only involved in osteoblastogenesis during ECO and not during IMO, and thus it is hypothesized that it may influence the chondrocyte-to-osteoblast transformation.^43^

Runx2 is a vital transcription factor for regulating chondrocyte hypertrophy and differentiation. Different studies evidenced that the lack of Runx2 inhibits chondrocytes from undergoing hypertrophy.^44^ Another important molecule is the core binding factor-β (CBF-β), a co-activator of Runx2, CCAAT/enhancer-binding protein-β (*C/EBP-β*), and activating transcription factor 4 (ATF4), which are critical mediators of hypertrophy in chondrocytes.^45^ Runx2 also contributes to Osterix (Osx) expression, an essential transcription factor required for osteoblastogenesis during ECO and IMO. Particularly, in ECO, Osx stimulates the expression of MMP-13, which is responsible for the calcification and degradation of cartilage templates.^35^

BMPs, which belong to the TGF-β family, are critical mediators during ECO, namely in the regulation of proliferation and differentiation of MSCs-derived chondrocytes.^46^ For instance, BMP-2, -4, and -7 have been shown to regulate the expression of Sox9 and stimulate ECO through the transcription of Runx2.^47^^,^^48^ Differently, BMP-3 can stimulate the maturation of terminal hypertrophic chondrocytes.^49^

The canonical form of the Wnt signaling pathway is also a key player in mediating the hypertrophic state of chondrocytes. Wnt canonical pathway controls the activation of β-catenin, which binds to lymphoid enhancer factor and T cell factor proteins. The resultant complex can promote the expression of Runx2, which induces hypertrophy. This phenomenon only occurs when the Wnt canonical pathway is activated; otherwise, β-catenin is degraded.^50^ In that scenario, chondrocytes will not be able to undergo hypertrophy, and consequently, a hyaline-like cartilaginous template is obtained, expressing high levels of Col II and aggrecan.^43^^,^^51^ Moreover, the Wnt pathway seems to be involved in regulating chondrocyte-to-osteoblast transformation because when the β-catenin gene was deleted from hypertrophic chondrocytes, bone formation substantially decreased.^52^ Inhibition or enhancement of this signaling pathway has been shown to control cartilage and bone formation in an on/off fashion.^53^^,^^54^ Furthermore, the activation of the Wnt pathway during the chondrocyte-to-osteoblast transformation was revealed to enhance bone formation.^53^

Notch signaling is another important pathway in the ECO process and is particularly relevant during hypertrophic maturation.^43^^,^^55^ Notch signaling pathway is triggered by cell-to-cell contact. When the receptor binds to ligands from hypertrophic chondrocytes, it leads to the proteolytic cleavage of the Notch receptor extracellular domain. Thus, it forms a complex with specific nucleus molecules, and consequently, the gene is transcripted. When the Notch pathway is inhibited during the final stages of ECO, the bone healing process is jeopardized, originating in shorter limbs composed of high populations of hypertrophic chondrocytes.^56^ Notch is also crucial for the preservation of osteochondroprogenitor’s multipotency.^55^ Likewise, the TGF-β signaling pathway can control chondrocyte proliferation and differentiation because it can suppress Runx2, stabilize Sox9, and induce the expression of BMP-2.^57^^,^^58^ In the perichondrium, FGF-18 and IGF-1 are produced to inhibit the proliferation of proliferative chondrocytes.^59^^,^^60^

## Design principles for endochondral tissue engineering

### Cell sources for bone formation

MSCs are the most used source of cells for bone engineering due to their fast proliferation, multipotency, self-renewal capacity, and ability to secrete a wide range of cytokines and growth factors.^18^^,^^61^ Such secreted bioactive molecules have anti-inflammatory and immunosuppressive effects, which allow MSCs to regulate inflammation while promoting tissue regeneration.^61^, ^62^, ^63^ One of the main advantages of using MSCs-based strategies is due to their immune privilege characteristic because they do not trigger an immune response.^64^^,^^65^ In addition, these cells have crucial roles in the ECO process. MSCs can easily differentiate into chondrocytes and osteoblasts, enhance cell proliferation and differentiation, and turn the microenvironment auspicious to chondrocytes to become hypertrophic.^66^ MSCs are present in several tissues, including bone marrow, adipose tissue, synovial membrane, periosteal, and umbilical cord.^18^^,^^67^ The selection process must consider the specificities of each tissue and the advantages/disadvantages of each isolation procedure. Bone marrow mesenchymal stem cells (BMMSCs) have been the most used source of cells in bone TE strategies, specifically those recapitulating the ECO process, due to their extensive characterization *in vitro* and well-established isolation protocols.^18^ During the healing process, BMMSCs are recruited to the injury site, indicating that these cells are highly sensitive to the release of bioactive molecules. In fact, these cells show appetence to respond to several molecules released after bone damage, inflammation, and bone repair.^18^ Another advantage is that bone marrow is a rich source of BMMSCs.^68^ However, the highly invasive harvesting procedure as well as the decline of the quantity and differentiation ability of BMMSCs with increasing donor age and *in vitro* passage are major disadvantages.^69^ BMMSCs-based TE strategies have shown the capacity to regenerate bone defects in several animal models. In most strategies, BMMSCs were applied in ECO approaches by differentiating into chondrocytes, achieving a hypertrophic phenotype *in vitro*, and then developing bone-like tissues *in vivo*.^19^^,^^22^^,^^25^^,^^70^, ^71^, ^72^, ^73^ For instance, ceramic scaffolds seeded with BMMSCs have been successfully tested in a clinical trial to repair large bone defects. Impressive results were obtained *in vivo*, as after 7 years, the implants showed good integration without any fracture.^74^

On the other hand, adipose-derived mesenchymal stem/stromal cells (ASCs) are becoming highly attractive in TE, mainly due to their ease of harvest and high abundance. In addition, isolating ASCs through the minimally invasive liposuction procedure adds value to a tissue that is normally discarded.^69^^,^^75^ ASCs have been incorporated into different scaffolds to construct bone grafts. For instance, osteogenically induced autologous ASCs seeded in scaffolds composed of polylactic acid coated with fibronectin^76^ or in a coral graft^77^ showed enhanced bone healing in rabbit and canine models, respectively. Interestingly, ASCs were also tested in a clinical trial by seeding them in β-tricalcium phosphate scaffolds combined with BMP-2 to develop a microvascular custom-made ectopic bone flap. The combination of ASCs with the osteoconductive scaffold allowed the development of well-ossified and vascularized constructs.^78^ ASCs combined with fibrin glue and cancellous bone grafts were also utilized to treat severe calvarial defects. Results showed deposition of new bone and almost complete calvarial continuity.^79^ More recently, fractionated human adipose tissue was obtained by washing and shuffling liposuction samples through syringes until obtaining small tissue particles. Instead of isolating ASCs, the particles were cultured in a proliferation medium combined with a dispersion of ceramic granules. Afterward, ASCs differentiated within the composites into hypertrophic chondrocytes. The composites were implanted in nude mice, resulting in reproducible bone and bone-marrow-like tissue.^80^

Umbilical-cord-derived stem/stromal cells (UCMSCs) have also been utilized for bone-engineered constructs due to their promising characteristics. The umbilical cord is a perinatal tissue rich in MSCs. These cells are at a development level between adult MSCs and embryonic stem/stromal cells (ESCs). One of the advantages is that collecting and banking UCMSCs after delivery has become a trendy procedure.^81^ In the past, the umbilical cord was considered a poor source of MSCs. However, the isolation protocols have been significantly improved, and nowadays, the umbilical cord is regarded as a promising source, allowing to obtain UCMSCs with high efficiency.^82^ The UCMSCs' immune properties have been tested, and it was found that they also have immunosuppressive properties and low immunogenicity, indicating that it may be possible to utilize them in allogeneic transplants.^83^, ^84^, ^85^, ^86^ UCMSCs demonstrate similar biological and therapeutic properties compared with BMMSCs and ASCs.^87^^,^^88^ Moreover, UCMSCs have increased immunomodulation ability compared with BMMSCs and ASCs.^84^ Comparisons between MSCs isolated from bone marrow, adipose tissue, and umbilical cord were performed by evaluating their morphology, easiness of isolation, colony frequency, expansion potential, ability to differentiate into multiple types of cells, and immune phenotype.^69^ Although no differences were found regarding the morphology and phenotype, ASCs and BMMSCs successfully differentiated into the classical triple lineage, whereas UCMSCs could not undergo adipogenic differentiation. In addition, the isolation yield of UCMSCs was the lowest (63%) compared to its counterparts (100%). In contrast, UCMSCs showed the highest proliferation potential.^69^

The dental pulp is another interesting source of MSCs that would otherwise be discarded during dentist interventions. Dental-pulp-derived MSCs (DPMSCs) present good angiogenic and osteogenic potential and were already applied in an *in vivo* ECO experiment.^89^, ^90^, ^91^, ^92^, ^93^ In that experiment, DPMSCs were seeded in collagen scaffolds and subsequently implanted in calvaria critical defects. DPMSCs were not induced to undergo chondrogenic differentiation before implantation. During a follow-up of three months, it was possible to observe DPMSCs undergoing chondrogenic differentiation, chondrogenic hypertrophy, and finally, developing new bone tissue.^92^ Periosteum-derived cells (PDCs) are another cellular source that has become of great interest for bone-engineered strategies. These progenitor cells present in the outer layer of bone structures are recruited during bone repair and have been shown to support both chondrogenesis and osteogenesis.^30^ Interestingly, PDCs present an improved bone regenerative capacity compared with BMMSCs *in vivo*. Moreover, PDCs show higher levels of proliferation, clonogenicity, and differentiation into chondrogenic lineages than bone marrow-derived counterparts.^94^ Due to the contribution of PDCs cells during the formation of the cartilaginous soft callus, they have been utilized for bone TE strategies. For instance, PDCs were used to create callus organoids *in vitro* until achieving an intermediate cartilage stage. Afterward, the organoids were seeded in an agarose mold, fused for 24 h, and implanted in murine critical-sized defects. X-ray and micro-CT scans exhibited the full bridge of the gap, resulting from the formation of cortical-like bone tissue with a nonmineralized compartment, suggesting the presence of a medullary cavity containing bone marrow.^95^ However, PDCs can present different profiles and properties depending on the anatomical locations. For instance, scaffolds seeded with tibial and mandibular PDCs were able to promote mineralization 8 weeks after ectopic implantation, whereas maxilla-derived PDCs did not.^96^

During the initial acute immune response, macrophages are recruited to the injury site and remain *in situ* until the end of the regeneration process. Macrophages are one of the first cells that arrive at the injury place, cleaning cell debris and protecting the tissue from pathogens.^97^ Furthermore, these immune cells produce growth factors to recruit other cell phenotypes to start natural bone regeneration. Macrophages are key elements in the bone regenerative process due to their plasticity. Macrophages' phenotype polarizes depending on the stimuli from the surrounding environment.^98^ During the early stages of bone repair, macrophages tend to assume the M1 pro-inflammatory phenotype. Following the normal regenerative process, macrophages switch to the M2 anti-inflammatory phenotype. Only an efficient and timely switch from the M1 to the M2 macrophage phenotype results in an appropriate production of molecular cues to support bone regeneration. More recently, the specific role of macrophages during ECO regenerative process has been studied. For instance, Schlundt et al. observed the effect of macrophages in the early and late stages of bone regeneration, namely during inflammation and ossification, respectively.^99^ The reduction of macrophages using clodronate liposomes *in vivo* resulted in no significant effects on the early stages of bone healing. However, the reduced number of macrophages affected the ECO, specifically the maturation of the chondrogenic stages toward the woven bone formation. Furthermore, it was concluded that M2 macrophages were essential to occur ECO. The stimulation of macrophages into a pro-healing M2 phenotype in a collagen scaffold with interleukin-4 (IL-4) and IL-13 improved bone formation *in vivo*, with significantly higher callus and bone volumes when compared with a control collagen scaffold only with PBS treatment.^99^

Another option for bone TE strategies is utilizing immature or differentiated progenitor cells, such as chondrocytes and chondroprogenitors, for endochondral bone repair. Porcine articular chondrocytes combined with polycaprolactone (PCL) scaffolds loaded with BMP-2 successfully recreated the ECO process *in vitro*. Upon implantation, bone formation occurred, but only in the periphery of the scaffold.^100^ Chick embryo chondrocytes were also employed to produce bone following *in vivo* implantation.^101^ Although chondrocytes are an appealing source of cells for ECO, they present limited proliferation and may become hypertrophic after implantation. Chondroprogenitor cells are also primed for chondrogenic differentiation, and their expansion does not modify differentiation.^102^

The endothelial progenitor cells (EPCs) can also be helpful for ECO strategies. EPCs, which are derived from mononuclear cells, can be applied to enhance vascularization during ECO.^103^ It is possible to obtain two distinct populations of EPCs: early and late EPCs.^104^ Usually, late EPCs are the most employed in TE strategies and are named after their late outgrowth potential. Interestingly, EPCs can potentially promote osteogenic differentiation of MSCs via the MAPK-dependent pathway.^105^ Peripheral blood CD34^+^ cells and fracture-hematoma-derived cells have also been considered appealing for bone regeneration. Matsumoto and colleagues transplanted these cells intravenously in athymic nude rats to verify if the fracture healing is supported by vasculogenesis and osteogenesis via regenerative plasticity of CD34^+^ cells. Results indicated that peripheral blood CD34^+^ cells enhance bone regeneration by ECO.^106^

Although several studies utilize only one type of cells to recreate the ECO process, co-culture systems have been applied to better mimic what happens in the native environment following a bone injury, where several types of cells are recruited and interact together to proceed with bone repair. Their crosstalk is crucial for successful bone regeneration to occur.^107^ For example, MSCs directly influence macrophage polarization and regulate part of the immune response. On the other hand, the proteins secreted by these immune cells regulate the behavior of MSCs and the progress of ECO.^62^^,^^66^ Co-culture systems are being adopted, for example, to overcome vascularization problems. As previously explained, MSCs play a crucial role in cell inter-communication during ECO, including during vascularization. The produced angiogenic factors by MSCs induce the differentiation of endothelial cells. Subsequently, endothelial cells release osteogenic factors that lead to MSCs differentiation.^108^ In an attempt to enhance bone formation, Correia et al. co-cultured human ASCs and human adipose-derived microvascular endothelial cells (hAMECs) in semi-permeable and liquefied capsules, with or without osteogenic supplementation. Results showed that osteogenesis was enhanced compared with monocultures of ASCs, even without supplemental osteogenic differentiation factors.^109^ Using the same system, *in vivo* implantation was performed with or without *in vitro* pre-differentiation of human ASCs.^110^ Interestingly, the co-culture system without any pre-differentiation obtained similar mineralization levels *in vivo* than the former pre-differentiated ones. Such results evidence that the cues secreted by endothelial cells successfully induced the osteogenic differentiation of ASCs, thus highlighting the importance of cell-cell direct contact and signaling. The same encapsulation system was also employed in two different approaches: the co-culture of ASCs with human primary osteoblasts and the triculture of UCMSCs, human umbilical vein endothelial cells, and macrophages.^13^^,^^17^ The results were similar, with better osteogenic differentiation and mineralization in cocultures and tricultures compared with monocultures.

### *In vitro* culture conditions to reproduce ECO process

In the literature, there is no consensus on the conditions, medium composition, and days of culture necessary to recapitulate *in vitro* the ECO process. The cell type chosen is an important topic that dictates the endochondral priming medium formulation. Usually, the chondrogenic differentiation of MSCs induces a certain degree of hypertrophy, although an additional *in vitro* hypertrophic priming step is typically applied.^42^^,^^111^ The chondrogenic differentiation medium is commonly composed of Dulbecco’s modified Eagle’s medium (DMEM) supplemented with penicillin/streptomycin, sodium pyruvate, L-proline, L-ascorbic acid 2-phosphate, linoleic acid, BSA, insulin-transferrin-selenium premix, and dexamethasone, along with various growth factors.^111^ Besides the chondrogenic medium, some growth factors are likewise crucial during early-stage and terminal MSCs differentiation and chondrogenic phenotype maintenance. For instance, the TGF-β superfamily is polyvalent, inducing chondrogenesis, proliferation, matrix deposition in MSCs, and hypertrophy, thus acting in all the essential stages of endochondral bone formation.^18^^,^^112^ Supplementing the culture medium with TGF-β2 and TGF-β3 seems to promote chondrogenesis, hypertrophy, and vascularization in MSCs. On the other hand, it is described that TGF-β3, together with β-glycerophosphate (β-GP), induces a higher level of mineralization than the combination of TGF-β1 and β-GP.^73^ TGF-β along with thyroid hormones in the culture medium for 14 days accelerate MSCs-derived chondrocytes entering into a hypertrophic state.^113^ Furthermore, BMPs have an active role in bone homeostasis. In particular, supplementation with BMP-6 increased the expression of Col X, one of the main ECO markers of hypertrophic chondrocytes.^114^

VEGF is a crucial factor in angiogenesis that directly influences ECO. During ECO, hypertrophic chondrocytes produce VEGF to recruit the host blood vessels that invade the diaphysis by the periosteal bud.^62^ The inhibition of VEGF expression results in lower neovascularization and modifies the behavior of hypertrophic chondrocytes, harming the bone repair process. Therefore, this growth factor is crucial for enhancing endochondral bone regeneration and vascularization.^115^ Furthermore, the presence of VEGF seems necessary to recruit MSCs to the injured site. VEGF is present in the platelet-rich plasma. Besides VEGF, platelet-rich plasma possesses several other growth factors, including PDGF, TGF-β, and IGF. Strategies using platelet-rich plasma can simultaneously improve angiogenesis and bone formation while showing improved results compared with VEGF.^116^ All the described constituents of platelet-rich plasma seem to have a beneficial effect on bone regeneration. PDGF has been shown to improve vascularization during bone regeneration, induce the proliferation of osteoblasts, and increase VEGF production by endothelial cells.^117^ When recombinant human PDGF was administered in Sprague-Dawley rats, higher bone density and strength could be observed, resulting in a bone regeneration improvement.^118^ IGF influences endothelial cell recruitment and tubular formation. However, its effect on vascularization during bone repair is unknown.^119^ The inhibition of IGF triggered the production of bone with lower density and shorter hypertrophic zones, evidencing its relation with bone development.^120^

Several ECO approaches utilize ascorbic acid (AA), β-GP, and dexamethasone in low amounts as supplements to achieve hypertrophy of MSCs-derived chondrocytes, as shown in Tables 1 and 2 (ECO scaffold-based and scaffold-free approaches). These three components are well-known osteogenic differentiation factors used in IMO approaches. Dexamethasone induces and regulates RUNX2 expression in hypertrophic chondrocytes. AA enhances Col I production by MSCs. β-GP provides phosphate for the mineralization stage, stimulating the production of hydroxyapatite, whereas its inorganic phosphate also regulates osteogenic pathways.^143^ Thyroxine, triiodothyronine, and IL-1β have also been employed to promote hypertrophic markers' expression or improve cartilage remodeling.^42^^,^^144^^,^^145^ Of note, previously to the hypertrophic stimulus, it is required that MSCs differentiate into chondrocytes to create the cartilaginous template. For that, MSCs are required to be in a 3D environment, which can be achieved by creating cell aggregates or seeding MSCs in 3D bioengineered matrices.^146^ Ultimately, MSCs are stimulated to differentiate into chondrocytes by *in vitro* culture for 2–4 weeks in a chondrogenic medium. Usually, mineralization occurs after three weeks of culture in a chondrogenic differentiation medium, followed by two weeks in a hypertrophic medium. The different *in vitro* and *in vivo* ECO models and the respective culture conditions described in the literature are summarized in Tables 1 and 2. Overall, there is no consensus in the literature considering the time required for the chondrogenic and hypertrophic *in vitro* stimuli as well as the supplementary factors necessary for each culture medium.

**Table 1:** ECO-scaffold-based approaches

Authors/Reference	Cell type	*In vitro* culture				*In vivo* study	Outcome
		Chondrogenic supplementation	Time	Hypertrophic supplementation	Time		
Marmotti et al.^81^	Umbilical cord MSCs	Chondrogenic differentiation Kit (EuroClone)	3 weeks	10mM HEPES Buffer, 1mM Na pyruvate, 1% ATB, 1% ITS-A, 4.7 *μ*g/mL linoleic acid, 1.25 mg/mL human serum albumin, 0.1 mM AA, 10^−8^ M DEX, 10 mM *β*-GP, 0.05 *μ*M L-thyroxin	2 weeks	—	After 3 weeks, the deposition of the cartilaginous matrix and a slight upregulation of *SOX-9* expression were observed. *CBFA-1* was upregulated in both endochondral and osteogenic control groups. After 5 weeks, *SOX-9* was downregulated, whereas CBFA-1 was upregulated.
Jukes et al.^121^	Embryonic stem cells	100 nM DEX, 50 μg/mL AA, 100 μg/mL sodium pyruvate, 40 μg/mL proline, and ITS-plus.	3 weeks	10^−7^ M retinoic acid (first 3 days), 0.2 mM AA, 2.5 μM compactin, and 0.01 M β-GP	3 weeks	3 weeks	After being implanted in nude mice, it was possible to observe a gradual change of cartilage turning into bone. Bone tissue seemed to have a similar structure to bone marrow. Researchers observed that isolated chondrocytes did not produce the same results as derived chondrocytes because no signs of ECO were found.
Thompson et al.^22^	Bone marrow MSCs	20 ng/mL TGF-β3, 50 mg/mL AA, 40 mg/mL Proline, 100 nM DEX, 1xITS, 0.11 mg/mL sodium pyruvate	3 weeks	1 nM DEX, 1xITS 1nM L-thyroxine, 50 mg/mL AA, and 10mM β-GF	2 weeks	8 weeks	CHyA and CHA ECO constructs enhanced *in vivo* vascularization. CHyA constructs presented the highest cartilage formation *in vitro* and the highest bone formation *in vivo* compared with CHA.
Daly et al.^122^	Bone marrow MSCs	100 U/mL penicillin, 100 μg/mL streptomycin, 100 μg/mL sodium pyruvate, 40 μg/mL L-proline, 50 μg/mL AA, 4.7 μg/mL linoleic acid, 1.5 mg/mL BSA, 1xITS, 100 nM DEX, 2.5 μg/mL amphotericin B, 500 ng/mL BMP-2 and 10 ng/mL TGF-β3	2 weeks	—	—	8 weeks	The bone deposition was not as high as in the positive control without microchannels. However, the scaffolds with microchannels showed an improved interaction with the host cells, enhanced vascularization, and hydrogel degradation.
Leijten et al.^123^	Bone marrow MSCs	10 ng/mL TGF-β3	5 weeks	—	—	5 weeks	Oxygen tension was revealed to be an important factor in directing cell differentiation. Only normoxia-preconditioned scaffolds showed matrix calcification, vascularization, and evidence of bone repair through ECO.
Mikael et al.^124^	Bone marrow MSCs	10 nM TGF-β1, 1xITS, linoleic-BSA, 50 mg/mL AA, 100 mg/mL sodium pyruvate, 40 mg/mL proline and 100 nM DEX	2 weeks	50 nM thyroxine, 7 mM β-GP, ITS+1, 50 μg/mL AA, 100 μg/mL sodium pyruvate, 40 μg/mL proline, and 0.01 μM DEX	2 weeks	8 weeks	The newly deposited extracellular matrix recruited host cells and enhanced bone regeneration.
Li et al.^125^	Bone marrow MSCs	100 nM DEX, 1% (v/v) ITS, 40 μg/mL AA, 50 μg/mL proline, and 10 ng/mL TGF-β3	2 weeks	100 ng/mL thyroxine, 1 nM DEX, 40 μg/mL AA, 1% (v/v) ITS, and 50 μg/mL proline	2 weeks	12 weeks	Although both coated- and noncoated PEG scaffolds almost completely repaired the defects, the coated-PEG scaffold reconstructed the medullary cavity while bonding the two sides of the defect. The ectopic bone formation experiments showed improved bone formation in coated scaffolds and higher expression of MMP13, which is known to induce angiogenesis.
Bai et al.^126^	Bone marrow MSCs	10^−7^ M DEX, 1% (v/v) ITS, 50 μM AA, 1 mM sodium pyruvate, 50 μg/mL proline, and 20 ng/mL TGF-β3	2 weeks	10^−8^ M DEX, 50 mM thyroxine, 250 μM AA, 1 mM sodium pyruvate, and 50 μg/mL proline	2 weeks	8 weeks	Mangiferin (20 and 100 mM) protected the hypertrophic chondrocytes and improved cell viability in hypoxia-induced conditions. The constructs were also implanted in mouse middle femoral defect models for 8 weeks. The *in vivo* experiments showed that mangiferin induced cells autophagy and enhanced the bone repair via ECO.
Ji et al.^127^	Bone marrow MSCs	—	—	—	—	9 weeks	The PCL/nHA scaffold successfully improved the mechanical features of the hydrogel. The BMMSCs-loaded constructs cultured in a Transwell plate with macrophages induced the production of angiogenic factors, such as VEGF, by the macrophages. Moreover, BMMSCs within the constructs, when cultured with macrophages, induced M2 macrophage polarization. Improved bone repair and tissue vascularization were observed *in vivo*. Gene expression analysis indicated that bone was being repaired via ECO.
Chen et al.^128^	Bone marrow MSCs	Low glucose DMEM, 584 mg/mL L-glutamine, 100 units/mL penicillin, 100 mg/mL streptomycin, 0.1 mM nonessential amino acids, 1 mM sodium pyruvate, 0.4 mM L-proline, 50 mg/mL AA, 150 mg/mL AA, 10 ng/mL TGF-β3, 100 nM DEX, and 1% ITS1	2 weeks	Serum-free high glucose DMEM, 7 mM β-GP, 10 nM DEX, and IL-1β (50 pg/mL)	1 week		BMMSCs were differentiated into chondrocytes, hypertrophic chondrocytes, or osteoblasts. The multiphenotypic cells were cultured in a poly(lactide-co-glycolide) (PLGA)-collagen hybrid mesh and then decellularized. BMMSCs were cultured in the ECM scaffolds, and results showed that hypertrophic ECM significantly enhanced osteogenic differentiation compared with the other conditions.
Scotti et al.^129^	Bone marrow MSCs	Serum-free chondrogenic medium	3 weeks	Serum-free hypertrophic medium, 50 nM thyroxine, 7.0 × 10^−3^M β-GP, 10^−8^M DEX, 2.5 × 10^−4^M AA and IL-1β (50 pg/mL)	2 weeks	12 weeks	BMMSCs were seeded in Col I scaffolds and sequentially cultured in chondrogenic and hypertrophic media. The strategy recapitulated the mature vasculature formation and developed a response to inflammatory signals and large bone marrow spaces.
Freeman et al.^23^	Bone marrow MSCs	HG DMEM, 10 ng/mL TGF-β3, 50 μg/mL AA, 4.7 μg/mL linoleic acid, 100 nM DEXA, and 1 × ITS	21 days	—	—	8 weeks	BMMSCs were seeded in PCL scaffolds. The constructs that were primed with chondrogenic differentiation medium induced more vessel recruitment throughout defects compared with intramembranous constructs; 50% of the animals treated with the endochondral constructs presented full bone union along the sagittal suture line.
Xie et al.^130^	Bone marrow MSCs	HG DMEM, 1% P/S, 1% sodium pyruvate, 1% ITS, 1 × 10^−7^ DEX, 50 μg/mL AA, and 10 ng/mL TGF-β3	3 weeks	—	—	4 weeks	BMMSCs were seeded in hydrogel microspheres (MSs) by digital light-processing (DLP) printing. The developed osteocallus organoids displayed stage-specific gene expression patterns that recapitulated the endochondral ossification process. In addition, the osteocallus organoids efficiently led to rapid bone regeneration within only 4 weeks.
Pitacco et al.^131^	Bone marrow MSCs	HG DMEM GlutaMAX, 100 U/mL penicillin, 100 μg/mL streptomycin, 100 μg/mL sodium pyruvate, 40 μg/mL L-proline, 50 μg/mL AA, 4.7 μg/mL linoleic acid, 1.5 mg/mL BSA, 1x ITS, 100 nM DEX, 10 ng/mL TGF-β3, and aprotinin at 5% pO2	3 weeks	50 ng/mL rh-BMP-2 (early hypertrophic) or 50 nM L-thyroxine, 100 nM DEX, 250 μM AA and 10 mM β-GP (late hypertrophic)	2 weeks		BMMSCs were incorporated into fibrin-based bioinks and bioprinted into PCL frameworks. After implantation, the constructs were rapidly remodeled into bone. Moreover, the early hypertrophic constructs supported higher vascularization and bone formation levels than the chondrogenic constructs.
Jeyakumar et al.^132^	Bone marrow MSCs	1% ITS, 1% BSA, 25 mM HEPES, DMEM high glucose, 100 nM DEX, 50 μg/mL AA, 10 ng/mL TGF-β1	2 weeks	1 nM DEX, 50 μg/mL AA, 50 ng/mL L-thyroxine, 10mM β-GP	6 weeks		BMMSCs were seeded in decellularized cartilage ECM containing silk fibroin. Results showed that the hybrid biomaterial significantly affected the hypertrophy-mediated osteogenic differentiation of stem cells. The early hypertrophy markers decreased, whereas the late hypertrophic markers increased.
Lin et al.,^133^	Bone marrow MSCs	HG-DMEM, 1% ATB, 0.1 μM DEX, 40 μg/mL L-proline, 1 × ITS, 50 μg/mL AA, and 10 ng/mL TGF-β3	14 days	HG-DMEM, 10% FBS, 1% ATB, 0.1 μM DEX, 10 mM β-GP, and 50 μg/mL AA.	14 days		ECO was recapitulated *in vitro* by including an endothelial cell network in a BMMSCs-ECM tissue. Firstly, the BMMSCs-ECM tissue was primed with chondrogenic and osteogenic factors and then embedded in a gelatin/fibrin hydrogel seeded with BMMSCs and endothelial cells. The tissue constructs promoted endothelial cell network generation *in vitro* and significant blood vessel recruitment *in vivo*.

β-GP, β-glycerophosphate; AA, Ascorbic acid; ATB, antibiotic-antimycotic; DEX, dexamethasone; DMEM, Dulbecco’s modified Eagle’s medium; FBS, fetal bovine serum; ITS, insulin-transferrin-selenium; TGF, transforming growth factor.

**Table 2:** ECO-scaffold-free approaches

Authors/Reference	Cell type	*In vitro* culture				*In vivo* study	Outcome
		Chondrogenic supplementation	Time	Hypertrophic supplementation	Time		
Wang et al.^134^	Bone marrow MSCs	Cyagen kit 100 mL/L DEX, 3 mL/L AA, 10 mL/L ITS, 1 mL/L sodium pyruvate, 1 mL/L proline and 10 mL/L TGF- β3	3 weeks	10^−8^ M DEX, 2.5 × 10^−4^M AA, 50nM thyroxine, 7 × 10^−3^ M β-GP	1 week	—	Results indicate that PEMF and hypertrophic supplementation may have caused the differentiation of hypertrophic chondrocytes into osteoblasts. This conclusion was reinforced by the high expression of osteogenic markers, namely BSP, Col I, and OSX. Even without the hypertrophic cues, 1 mT PEMF also presented a high expression of these markers.
Sasaki et al.^135^	Bone marrow MSCs	—	—	10^−2^ M β-GP, 50 mg mL^−1^ AA and 1 × 10^−6^ M DEX	3 weeks	—	3D *in vitro* model to evaluate the angiogenic induction of the ECO process. Chondrogenic, hypertrophic, and osteogenic markers were evaluated. Cartilaginous aggregates showed a mineralized core, resembling the beginning of ECO. OPN was present but not OCN.
Liu et al.^25^	Bone marrow MSCs	1% ATB, 1% ITS, 100 nM DEX, 50 μM AA, 23 μM L-proline, and 10 ng/mL TGF-β3	4 weeks	1% ATB, 10% FBS, 5mM β-GP, 10 nM DEX, and 50 μg/mL AA	4 weeks	4 weeks	Chondrogenic, hypertrophic, and osteogenic markers were evaluated during the following 56 days. ECO constructs showed the highest expressions of chondrogenic, hypertrophic, and osteogenic markers. Moreover, samples were positioned between two flat platens connected to a mechanical sensor. Compressive forces were recorded up to 10% strain. The ECO-based MSCs-laden constructs presented a significantly higher Young’s modulus than controls.
Zhang et al.^136^	iPSCs	10 ng/mL BMP-4, 10 ng/mL TGF-β3, 100 nM DEX, 50 μg/mL AA, 100 μg/mL sodium pyruvate, 40 μg/mL proline, and ITS-plus	2 weeks	10 ng/mL BMP-4, 1 nM DEX, 50 μg/mL AA, 100 μg/mL sodium pyruvate, 40 μg/mL proline, ITS-plus, 50 ng/mL thyroxine and 20 mM β-GP	2 weeks	8 weeks	No teratoma was observed. The cartilaginous pellets showed an enhanced expression of chondrogenic-related genes, decreased expression of pluripotent genes, and deposition of cartilage ECM. Vascularized bone tissue was observed. Mechanical stimulation was also analyzed, and the results suggest that the shear stress enhances iPSCs differentiation into the mesodermal lineage through the Bmp-4-Smad signaling pathway.
Huang et al.^137^	Human adipose tissue	10 ng/mL TGF-β3, 10 ng/mL BMP-6, 10^−5^ M AA, and 10^−7^ M DEX	4 weeks	0.01 M β-GP, 10^−7^ M DEX, and 10^−5^ M AA	2 weeks	12 weeks	Hypertrophic cartilaginous constructs were obtained from human lipoaspirates. After implantation in rats’ calvarial defects, the hypertrophic cartilaginous constructs were shown to support bone regeneration via ECO.
McDermott et al.^138^	Bone marrow MSCs	1% ITS + Premix, 1 mM sodium pyruvate, 100 μM nonessential amino acids, 100 nM DEX, 0.13 mM AA, and 1% P/S	48 h	—	—	12 weeks	The mechanical loading improved endochondral ossification bone repair; however, tissue vascularization was jeopardized.
Osinga et al.^139^	Adipose-derived MSCs	10 ng/mL TGF-β3, 10^−7^ M DEX, 0.01 mM AA, and 10 ng/mL BMP-6	4 weeks	0.01 M β-GB, 10^−8^ M DEX, 50 mM/L thyroxin, and 50 pg/mL IL1-β	2 weeks	8 weeks	ASCs aggregates were cultured sequentially in chondrogenic and hypertrophic media. After subcutaneous implantation, a successful ECO could be noticed, including bone-like ECM formation, proper integration with the host vasculature, and the presence of bone marrow components.
Dang et al.^140^	Bone marrow MSCs	10% ITS + Premix, 100 nM DEX, 37.5 μg/mL AA, 1 mM sodium pyruvate, 100 μM nonessential amino acids, 10 ng/mL TGF-β1	2 weeks	100 ng/mL BMP-2	3 weeks	—	A controlled drug delivery system was created to control chondrogenic and osteogenic differentiation. Compared with the control, the system generated could accelerate chondrogenesis and osteogenesis at week 2. At the end of week 5, these aggregates showed higher mineralization levels and bone markers.
Hall et al.^95^	Human periosteum-derived cells PDCs	LG-DMEM, 1% antibiotic-antimycotic, 1 × 10^−3^ M AA, 100 × 10^−9^ M DEX, 40 μg mL^−1^ proline, 20 × 10^−6^ M of Rho-kinase inhibitor Y27632, ITS + Premix, 100 ng mL^−1^ BMP-2, 100 ng mL^−1^ growth/differentiation factor 5 (GDF5), 10 ng mL^−1^ TGF-β1, 1 ng mL^−1^ BMP-6, and 0.2 ng mL^−1^ basic FGF-2	21 days	—	—	8 weeks	Human periosteum-derived cells were utilized to generate microspheroids that are differentiated into callus organoids. The created callus organoids spontaneously assembled *in vitro* into large engineered tissues. The mature microspheroids were chondrogenically differentiated toward hypertrophy. After *in vivo* implantation in a critical-sized defect, results demonstrated presence of mineralization after 2 weeks, and bridging of defects was detected after 4 weeks followed by increased corticalization until week 8.
Hall et al.^141^	Human periosteum-derived cells	LG-DMEM, 1% antibiotic-antimycotic, 1 × 10^−3^ M AA, 100 × 10^−9^ M DEX, 40 μg mL^−1^ proline, 20 × 10^−6^ M of Rho-kinase inhibitor Y27632, ITS + Premix, 100 ng mL^−1^ BMP-2, 100 ng mL^−1^ GDF5, 10 ng mL^−1^ TGF-β1, 1 ng mL^−1^ BMP-6, and 0.2 ng mL^−1^ basic FGF-2	21 days	—	—	4 weeks	This work proposes a proof-of-concept on the feasibility of image-guided robotic biomanufacturing of spheroid-based implants. After cartilaginous spheroids design and assembly, they were implanted subcutaneously in order to investigate the influence of spheroid fusion parameters on endochondral ossification.
Herberg et al.^142^		HG-DMEM, 1% ITS + Premix, 1 mM sodium pyruvate, 100 μM nonessential amino acids, 100 nM DEX, 0.13 mM AA, and 1% P/S	8 days	—	—	12 weeks	BMMSC tubes loaded with TGF-β1 and BMP-2 were proposed to recapitulate ECO. BMMSCs sheets were used as control. After orthotopic implantation, 75% of the defects implanted with BMMSCs tubes were bridged by week 12, whereas BMMSCs sheets yielded only 33% bridging.

β-GP, β-glycerophosphate; AA, Ascorbic acid; ATB, antibiotic-antimycotic; DEX, dexamethasone; DMEM, Dulbecco’s modified Eagle’s medium; FBS, fetal bovine serum; ITS, insulin-transferrin-selenium; TGF, transforming growth factor.

### Current tissue engineering approaches: How to achieve endochondral ossification *in vitro* and *in vivo*

**Figure 3:**
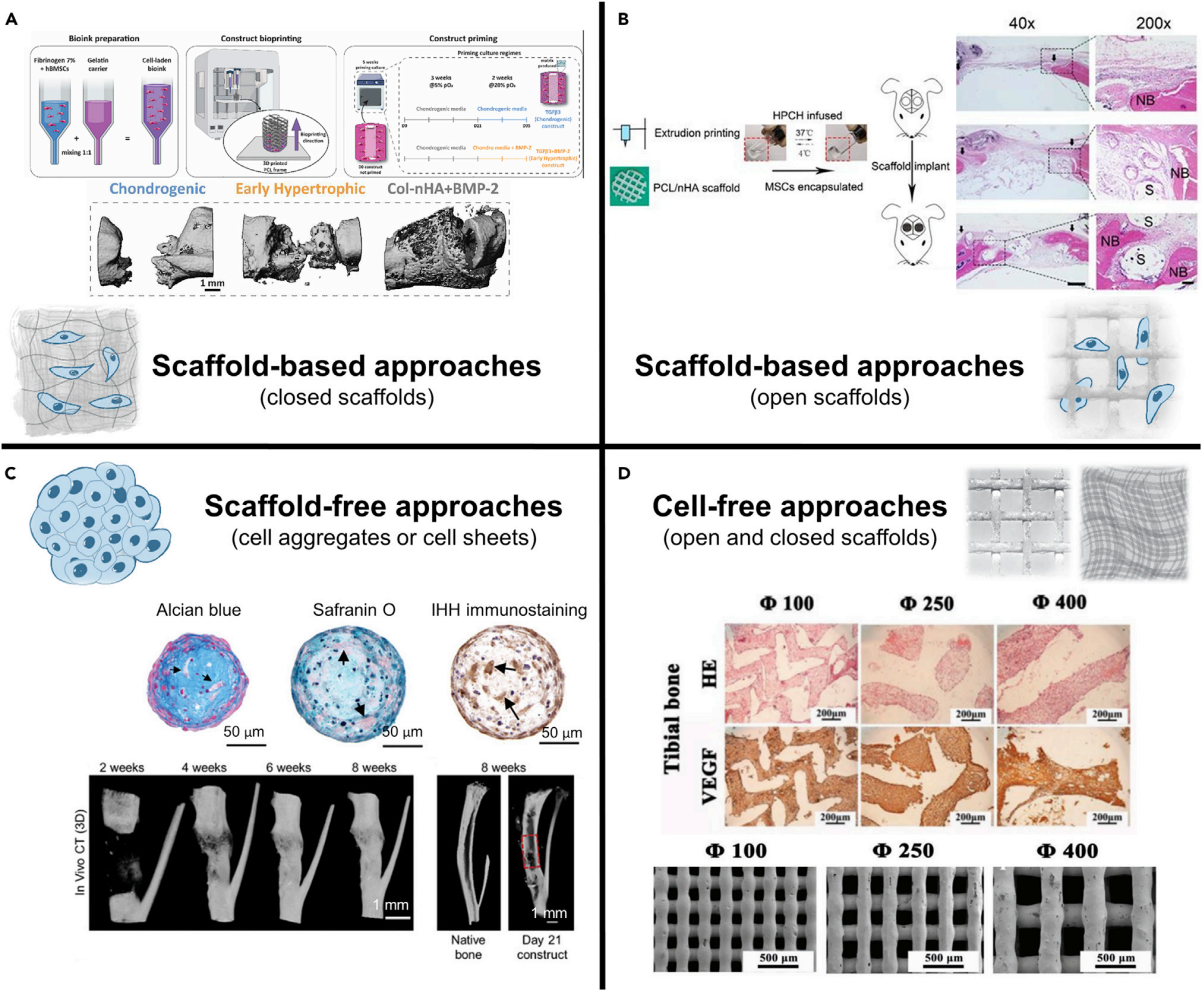
Examples of different ECO-based strategies (A) Schematic representation of the bioprinted cartilaginous template fabrication process. Micro-CT analysis of bone formation in the defects after 12 weeks of implantation. Scale bar: 1 mm. Reprinted with permission.^131^ Copyright 2022, Elsevier. (B) Schematic representation of the fabrication of the hybrid scaffold and *in vivo* implantation. Representative photomicrographs of hematoxylin and eosin staining of calvarial defects at 9 weeks post-surgery. The control, PCL/nHA, and PCL/nHA + HPCP groups, respectively, are represented. NB, new bone; S, scaffold. Scale bars: 500 μm in 40X, and 100 μm in 200X. Reprinted with permission.^127^ Copyright 2020, Ivyspring International Publisher. (C) Representative sections of callus organoids. Nano-CT 3D rendering images over time of defect and cross-section of 3D rendering of the native tibia. Scale bars: 50 μm (top); 1 mm (bottom). Reprinted with permission.^95^ Copyright 2019, Wiley-VCH. (D) H&E and immunohistochemical staining of VEGF of the scaffolds in tibial defects after 2 weeks of surgery. Porous structure of plotted β-TCP scaffolds with different pore size. Scale bars: 200 μm (top); 500 μm (bottom). Reprinted with permission.^147^ Copyright 2019, Wiley-VCH.

**Figure 4:**
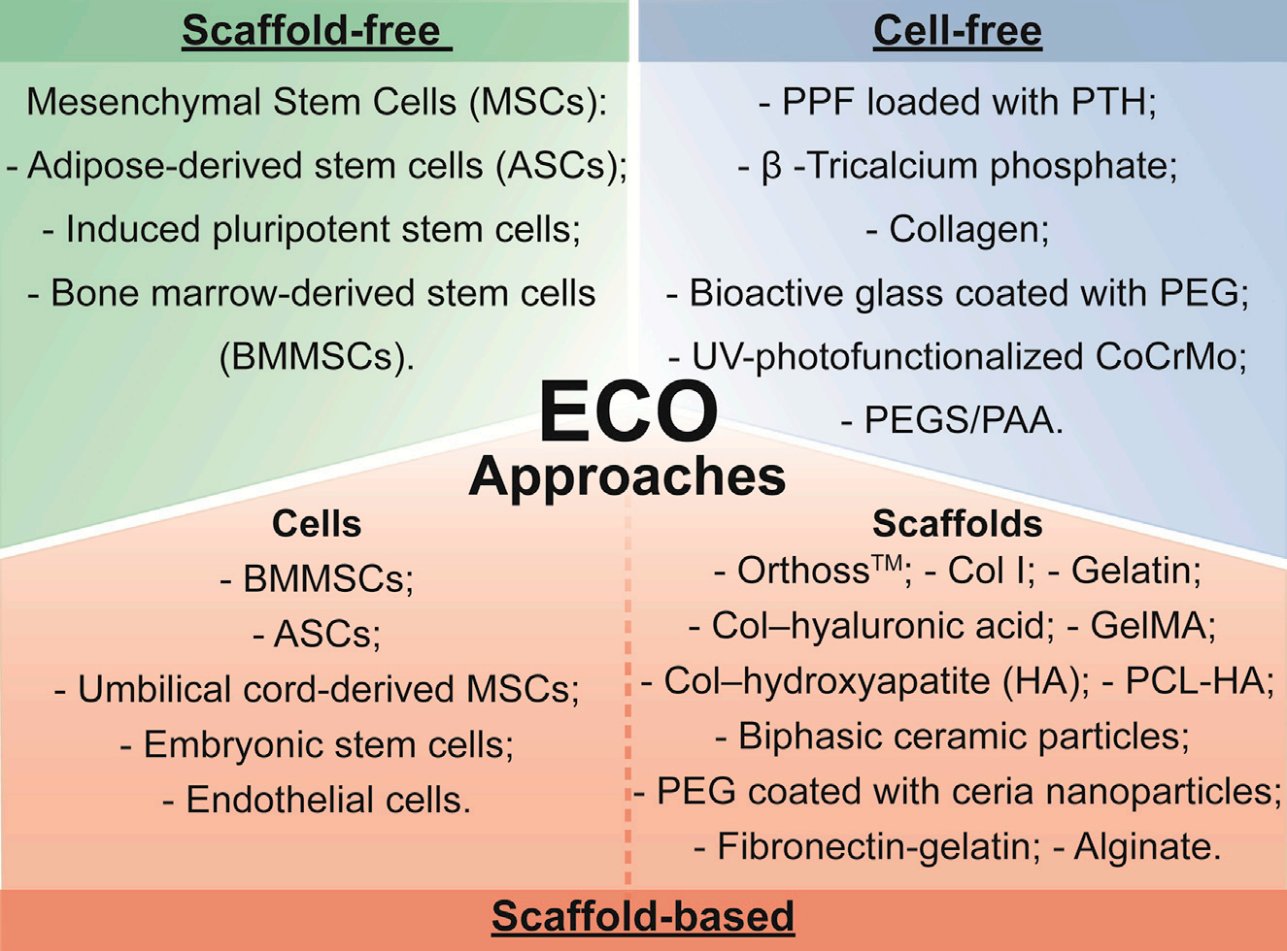
Representation of the most utilized cell types and biomaterials in the different ECO approaches Division of the ECO approaches could be categorized according to their use of cells (scaffold-free approaches), biomaterials (cell-free approaches), or a combination of both (scaffold-based approaches). Nomenclature: PPF, poly(propylene fumarate); PTH, parathyroid hormone; CoCrMo, cobalt-chromium-molybdenum alloy; PEGS/PAA, poly(glycerol sebacate)-co-poly (ethylene glycol)/polyacrylic acid.

Building an ECO strategy is a complex process with many variants. There is a need to select the type of cells, the proper supplementation, the composition of the scaffold, as well as if the study will be performed *in vitro* or *in vivo*. Furthermore, the strategy can combine materials and cells, named scaffold-based approaches, or utilize them individually, entitled cell-free and scaffold-free approaches (Figure 3). In the last decades, increasing TE strategies have been envisioned to mimic the ECO process. Regardless of the differences between the scaffold-based, cell-free, and scaffold-free approaches, the TE construct must provide biochemical, mechanical, or structural cues to promote tissue repair after implantation.^148^ In fact, the design of TE strategies should consider essential features for proper bone tissue regeneration, including biocompatibility, biodegradability, osteoinductivity, osteoconductivity, and appropriate surface properties.^75^ Figure 4 identifies the cell types and biomaterials more employed in each strategy.

#### Scaffold-based approaches

The most common strategy in TE relies on combining materials and cells to repair damaged tissue. During this review, this type of technology was named as scaffold-based approach. In the TE field, creating a 3D structure closer to the native tissue environment is vital to control cell behavior. However, the ECM produced by cells can be insufficient; thus, scaffolds are usually employed to support cell attachment, proliferation, and differentiation. In addition, scaffolds fill the tissue defect and support the surrounding tissue when implanted.^149^^,^^150^ The scaffolds should allow the exchange of gases, nutrients, and growth factors, to increase the cell survival rate.^74^ In addition, the ability of the implanted biomaterial to recruit and allow the invasion of the host cells and blood vessels are of utmost importance. When the focus is bone regeneration, the factors secreted by hypertrophic chondrocytes are what distinguish ECO from IMO repair. These factors induce blood vessel invasion, the recruitment of more cells and factors, and the differentiation of MSCs into osteocytes. Therefore, it is vital that the features of a scaffold, namely the porosity, interconnection of the pore network, topography, and geometry, are designed to allow penetration by the host’s blood vessels while driving recruited cells' differentiation following implantation.^151^, ^152^, ^153^

Collagen is one of the most used polymers in ECO because cartilaginous and osteogenic matrices are rich in Col I. Ultrafoam, a commercially available Col I mesh, seeded with ASCs, was already successfully utilized in ECO approaches.^80^ NuOss, CopiOs, Bio-Oss, Collagraft, and Vitoss are also commercially available collagen scaffolds tested for bone repair by ECO. These scaffolds contain calcium phosphate particles with natural hydroxyapatite (NuOss, Bio-Oss), 35% β-tricalcium phosphate (β-TCP), 65% synthetic hydroxyapatite (Collagraft), only β-TCP (Vitoss), or dibasic calcium phosphate (CopiOs).^154^ For instance, BMMSCs were seeded in Col I scaffolds and sequentially cultured in chondrogenic and hypertrophic media to recapitulate ECO process.^129^ The constructs were then implanted in nude mice for 12 weeks. Interestingly, such a strategy was able to mimic the typical processes associated with bone development, mature vasculature formation, response to inflammatory signals, and large bone marrow spaces capable of hosting hematopoietic stem cells.^129^ In a different strategy, BMMSCs were cultured in two different scaffolds: collagen–hyaluronic acid (CHyA) and collagen–hydroxyapatite (CHA). Constructs were sequentially cultured in chondrogenic and hypertrophic media and then implanted in bone defects of immune-competent Fischer F344 rats. Both ECO constructs enhanced *in vivo* vascularization, which might be explained due to the secretion of pro-angiogenic factors. CHyA constructs presented the highest cartilage formation *in vitro* and the highest bone formation *in vivo*.^22^ Besides collagen, other types of biomaterials are being proposed. For instance, nano-hydroxyapatite (nHA) and PCL were bioprinted to create a hybrid scaffold that improves the mechanical properties of chitin-derived hydrogels (Figure 3B). BMMSCs were loaded into a hydroxypropyl chitin hydrogel (HPCH), and then the hydrogel was perfused into the 3D-printed scaffold of PCL/nHA. The control condition consisted of constructs with 3D-printed scaffolds without the HPCH. The compression tests demonstrated that the PCL/nHA scaffold successfully improved the mechanical features of the hydrogel. The interactions between RAW264.7 macrophages and the BMMSCs-loaded hybrid construct were also evaluated. Briefly, BMMSCs within the hybrid construct induced the polarization of macrophages into an M2 phenotype, which showed the ability to recruit rat-derived ECs *in vitro*. RT-PCR analysis showed an enhanced expression of VEGF by the macrophages and their polarization into a pro-healing phenotype. Afterward, the constructs were implanted in calvarial defects of Sprague-Dawley rats for 9 weeks. Micro-CT, H&E, and Masson’s trichrome staining results showed improved bone repair and tissue vascularization using the constructs compared with the controls. Remarkably, both chondrogenic, hypertrophic, and osteogenic gene expression indicated that bone tissue was being repaired via the ECO approach, although cells were not previously primed into the chondrogenic lineage *in vitro*.^127^

The inflammatory response following the implantation of scaffolds can represent an obstacle for an appropriate bone tissue repair to occur. Therefore, biomaterials with anti-inflammatory properties are an interesting strategy, which has been particularly explored in ECO strategies. To explore this subject, polyethylene glycol (PEG) scaffolds coated with ceria nanoparticles were utilized to create an ECO strategy.^125^ Ceria nanoparticles are tolerable and effective against inflammation. Therefore, BMMSCs were seeded in these scaffolds and subsequently induced to differentiate into the chondrogenic lineage, followed by a hypertrophic lineage. Afterward, such constructs were implanted in nude mice and Friend leukemia virus B/N mice for 12 weeks to obtain ectopic bone formation and *in situ* bone repair. Constructs were analyzed by micro-CT and different histologic stainings and compared with noncoated PEG scaffolds. Although both scaffolds showed an appropriate restoration of the defects, only coated scaffolds could reconstruct the medullary cavity while bonding the two sides of the defect. The ectopic bone formation experiments showed an improved bone formation in coated scaffolds, as well as a higher expression of MMP13, which is known to induce angiogenesis.^125^

Autophagy of BMMSCs-derived chondrocytes is beneficial for these cells because it protects them from oxidative stress while promoting chondrocyte hypertrophy. Thus, an ECO approach utilized mangiferin, an antioxidant compound, combined with BMMSCs to understand if autophagy and chondrocyte hypertrophy were promoted. The BMMSCs were induced to differentiate into the chondrogenic lineage for 2 weeks and then seeded in a demineralized bone matrix. Afterward, cells were induced to differentiate into hypertrophic chondrocytes for 2 weeks, with and without (control) mangiferin treatment. Such constructs were implanted in mouse middle femoral defect models for 8 weeks. Initially, to test if the mangiferin was able to protect cells from the effects of the hypoxia condition, treated and nontreated hypertrophic chondrocytes in the demineralized bone matrix were exposed to cobalt chloride to mimic the hypoxia-induced injuries *in vitro*. Results showed that mangiferin (20 and 100 mM of concentration) protected the hypertrophic chondrocytes and improved cell viability in hypoxia-induced conditions. The *in vivo* experiments demonstrated that mangiferin promoted AMPK phosphorylation and, consequently, induced cell autophagy while enhancing bone repair via ECO.^126^

The 3D bioprinting technology was also proposed to promote ECO and improve vascularization. Pitacco and colleagues bioprinted cartilaginous templates to assess their ability to repair critical-sized bone defects.^131^ BMMSCs were loaded into fibrin-based bioinks and bioprinted into a PCL frame to reinforce the engineered constructs (Figure 3A). After chondrogenic priming, the constructs were implanted subcutaneously in nude mice for 12 weeks. Results demonstrated that chondrogenic differentiation was necessary to occur mineralization. Afterward, the constructs were submitted to a second priming in order to produce an early hypertrophic state. Ultimately, these chondrogenic and early hypertrophic constructs were implanted in a 5-mm rat femoral defect and were compared with a positive control (collagen—nHA scaffold, loaded with BMP-2). Results demonstrate that the early hypertrophic priming supported higher levels of vascularization and spatially distinct patterns of new formation compared with the positive control.^131^

In another study, 3D constructs of gelatin methacryloyl (GelMA) hydrogel containing BMMSCs and microchannel interconnected networks were printed using a sacrificial pluronic ink. Constructs without microchannels and untreated animals were used as control. During the first 2 weeks, scaffolds were exposed to 5% partial pressure of oxygen and then to 20% partial pressure of oxygen. Ultimately, the scaffolds were implanted in critical-sized defects of immune-competent Fischer rats. Results showed that the partial pressure of oxygen was important to improve the bone healing process compared with untreated scaffolds. Interestingly, the constructs without microchannels (solid templates) supported the highest levels of total bone formation. However, osteoclast/immune cell invasion, hydrogel degradation, and vascularization was only achieved in the presence of microchannels.^122^ Indeed, hypertrophic chondrocytes in the native tissue are exposed to hypoxia conditions; however, most of the hypertrophic chondrocytes used in ECO approaches are differentiated from MSCs cultured in normal oxygen conditions. This circumstance often leads to a decrease in the survival rate of the cells.^126^ In another study, BMMSCs aggregates were stimulated in a chondrogenic medium for 35 days using two different oxygen tensions, 2.5% O_2_ (hypoxia) or 21% O_2_ (normoxia).^123^ Results revealed that normoxia enhanced hypertrophy *in vitro*, whereas hypoxia resulted in hyaline cartilage formation and expression of hypertrophy inhibitors. The BMMSC aggregates were encapsulated in sodium alginate hydrogels and implanted in mice for 5 weeks. Hydrogels with hypoxic treatment maintained an avascular cartilage-like ECM, whereas those subjected to normoxia were highly invaded by the host vessels. These studies demonstrate that it is possible to program MSCs metabolically by providing different oxygen tensions. Importantly, these approaches can effectively direct chondrogenic differentiation and develop permanent articular cartilage or hypertrophic cartilage to be later implanted and support ECO.^122^^,^^123^ Taken together, high levels of hypoxia improve chondrogenic differentiation of MSCs, upregulating Col II and aggrecan markers while suppressing chondrogenic hypertrophy and downregulating the expression of Col X, MMP-13, and ALP.^51^

Another possibility to employ a close-to-native ECM is to use decellularized ECM. BMMSCs were cultured in the appropriate differentiation culture media to obtain chondrocytes, hypertrophic chondrocytes, or osteoblasts. Subsequently, the multiphenotypic cells were cultured in a poly(lactide-co-glycolide) (PLGA)-collagen hybrid mesh and then decellularized to get the respective ECM. Afterward, BMMSCs were cultured in the ECM scaffolds. Results showed that hypertrophic ECM significantly enhanced osteogenic differentiation compared with the other conditions. However, such conditions also presented the lowest cell proliferation levels.^128^

The scaffold-based approaches were pioneers in overcoming the issues related to cell delivery. This type of approach joins the advantage of using cells while avoiding the dispersion of the core contents to peripheral regions of the body after implantation. In addition, the biomaterials can prime the seeded cells to undergo differentiation. For instance, ECO approaches that utilize scaffolds usually intend to accelerate the differentiation process of MSCs. Therefore, the materials may present biochemical and mechanical cues that will guide cell differentiation and controlled-delivery systems of nutrients and growth factors, which can also make these approaches more self-sufficient. Nevertheless, minimizing the amount of implanted biomaterials and cells may be the next generation of TE strategies. The “minimalistic-engineering” approaches claim that a biomaterial should guide the performance and recruitment of the host’s cells to the defect site to balance the regenerative niche toward the healing process.^155^ Table 1 highlights promising examples reported in the literature of scaffold-based strategies aiming ECO repair.

#### Scaffold-free approaches

The scaffold-free approaches include all the strategies that do not need cell adherence to a biomaterial.^156^ This strategy is used when cells are able to produce enough ECM to support themselves. Usually, cells are employed as cell aggregates, tissue strands, or cell sheets. Furthermore, these building blocks should be able to merge and form larger structures.^157^^,^^158^ One of the main advantages of using MSCs-based strategies is that cells can continuously provide an active resource of several cytokines and growth factors, eliminating the need for supplemental bioactive molecules.^18^^,^^62^ On the other hand, the clinical translation of these strategies may be compromised because they rely on a series of *in vitro* cell manipulation techniques.^159^ Some scaffold-free examples have been proposed to recapitulate the ECO process. For instance, ASCs aggregates were cultured sequentially in chondrogenic and hypertrophic media for different periods. After subcutaneous implantation in CD1 nu/nu nude mice for 8 weeks, a successful ECO could be noticed, including bone-like ECM formation, proper integration with the host vasculature, and the presence of bone marrow components.^139^ In another strategy, BMMSCs cultured in a proliferation medium with AA for 10 days and then exposed to 0.25% trypsin-EDTA solution for 5–7 min achieved the deposition of a denser and well-structured ECM by promoting the deposition of insoluble collagen and the ECM contraction. The cell aggregates created by this protocol were sequentially cultured in a chondrogenic and osteogenic medium for 4 weeks each, to reproduce the ECO process. Samples were also cultured for 8 weeks in an osteogenic medium, representing the IMO approach (control). ECO constructs exhibited the highest expression of chondrogenic, hypertrophic, and osteogenic markers during all the experiments. Interestingly, ECO constructs showed higher Young’s modulus compared with IMO. It was also possible to understand that at the beginning of the *in vitro* ECO process, the MSCs condensation was mediated by N-cadherin.^25^ Wang and colleagues applied pulsed electromagnetic field (PEMF) stimuli to develop an *in vitro* chondrogenic and hypertrophic differentiation model.^134^ Firstly, rat BMMSCs were cultured in a 3D pellet culture system using a chondrogenic medium and were divided into three groups. Such groups were exposed to different intensities of PEMF (1, 2, or 5 mT) for 4 weeks. The hypothesis was that PEMF induces MSCs-derived chondrocytes to undergo hypertrophy without supplemental factors added to the culture medium. The three different PEMF intensities did not affect cell proliferation but seemed to decrease the maintenance of the cartilaginous template and accelerate the ECM degradation. Notably, 1 mT PEMF samples showed the highest expression of the hallmark of hypertrophic cartilage Col X compared with the other groups and the control (chondrogenic supplementation and no PEMF stimuli). Regarding the osteogenic evaluation, 1 mT PEMF condition resulted in higher expression of the BSP, Col I, and OSX markers in both experimental groups, with and without hypertrophic cues. These results showed that the last step of ECO, which means the formation of a bone-like tissue, was promoted even without any hypertrophic cues. Nevertheless, it would have been important to analyze the behavior of endothelial cells in these conditions because the release of endothelial factors characterizes ECO approaches.^134^ A controlled drug delivery system was created to promote ECO without the need for supplementation factors.^140^ For that, BMMSCs aggregates were cultured in chondrogenic medium supplemented with TGF-β1 and subsequently in osteogenic medium supplemented with BMP-2. GAGs and calcium content evaluation showed that 2 weeks of chondrogenic supplementation and 3 weeks of osteogenic supplementation led to improved chondrogenic and osteogenic differentiation levels. Then, TGF-β1 and BMP-2 were loaded in gelatin microparticles (GM) or mineral-coated hydroxyapatite microparticles (MCM), respectively, to test their release behavior. Researchers knew that TGF-β1 released by GM was faster due to previous studies.^160^^,^^161^ The control group consisted of cell aggregates cultured for the first 2 weeks with chondrogenic medium supplemented with TGF-β1, then 3 weeks with osteogenic medium supplemented with BMP-2, by exogenous and repeated supplementation. Compared with the control, the system generated could accelerate chondrogenesis and osteogenesis at week 2, as proved by GAG quantification and ALP activity analysis. At the end of week 5, these aggregates showed higher mineralization levels and bone markers (calcium, Col I, OPN, OCN). Without requiring the repeated supplementation of the culture media, this system represents a solution to achieve faster implantation. Whereas others took 7 to 8 weeks to induce ECO, this model successfully reduced cell culture time to 5 weeks^140^ Another group developed BMMSCs pellets with local morphogen presentation. TGF-β1 was used to stimulate the condensation of BMMSCs. Then, the scaffold-free approach was implanted in critical-sized bone defects in rat femora for 12 weeks, utilizing mechanical loading as a cue to recapitulate ECO repair. Interestingly, the micro-CT and histological staining assays revealed that mechanical loading improved endochondral ossification bone repair; however, it decreased tissue vascularization.^138^

Few 3D models are replicating the ECO process *in vitro*. Sasaki et al. utilized BMMSC (bulb/c mouse), scaffold-free constructs cultured under hypoxic conditions to reproduce the ECO process. The constructs were built by seeding the cells in the holes of a thermoresponsive poly-N-isopropylacrylamide hydrogel. After 12 h of culture, the constructs could be collected by decreasing the temperature from 37°C to room temperature. Then, 3D constructs were cultured in hypoxia conditions and under osteoinductive stimulation factors to promote chondrogenesis and osteogenesis, respectively. Chondrogenic, hypertrophic, and osteogenic markers were evaluated for up to 50 days. Alcian blue, Col II, and Col X staining proved the cartilaginous nature of the constructs after 20 days of culture. Then, Von Kossa staining allowed to identify the formation of a mineralized core after 30 days of culture, which was identified as hydroxyapatite. In addition, an *in vitro* angiogenesis model was utilized to study the angiogenic potential of the proposed constructs. Results indicated that the construct had specific angiogenic activity depending on its maturity level.^135^ Although the development of a 3D model replicating the ECO process could be a valuable tool to replace a portion of the high-cost *in vivo* experiments, the full replication of bone repair’s physiological and morphological aspects is still challenging and far from reality.

Induced pluripotent stem cells (iPSCs) are an interesting and increasingly used source of cells in TERM applications. A scaffold-free strategy employed iPSCs pellets to obtain bone repair by ECO. First, iPSCs were induced to differentiate into mesoderm germ layer cells. Subsequently, iPSCs were cultured in a chondrogenic differentiation medium, followed by a hypertrophic differentiation medium, both for 2 weeks in dynamic conditions. Ultimately, iPSCs pellets were implanted in a cranial bone defect model of Sprague-Dawley rats for 8 weeks. The tumorigenicity of the chondrogenic pellets was analyzed *in vitro* and *in vivo,* where no teratoma was observed. The cartilaginous pellets showed an enhanced expression of chondrogenic-related genes, decreased expression of pluripotent genes, and the deposition of cartilage ECM. ECO repair was analyzed by micro-CT and by histological and immunohistochemistry staining, evidencing well-vascularized bone tissue *in vivo*.^136^ PDCs are also attractive to develop ECO-based strategies. After generating callus organoids *in vitro* using PDCs, the organoids were implanted in murine critical-sized defects. Results exhibited the full bridge of the gap (Figure 3C), resulting from the formation of cortical-like bone tissue with a nonmineralized compartment, suggesting the presence of a medullary cavity containing bone marrow.^95^ Table 2 highlights promising examples reported in the literature of scaffold-free strategies aiming ECO repair.

#### Cell-free approaches

Cell-free approaches only use biomaterial architecture and composition to induce ECO. Without cell transplantation, cell-free approaches present fewer disadvantages regarding *ex vivo* cell manipulation, the risk of developing tumors, and ethical discussion.^162^ On the other hand, these approaches have to demonstrate the ability to recruit the host cells to the injury place and allow their differentiation *in situ*.^124^^,^^163^ Some strategies incorporate bioactive factors to induce cell attachment, bone ingrowth, or blood vessel recruitment. Wojda and colleagues created a local delivery system of parathyroid hormone (PTH) to stimulate bone regeneration.^164^ Several quantities of PTH (0, 1, 3, 10, or 30 μg) were loaded in a thiol-ene hydrogel and then polymerized in and around a poly (propylene fumarate) (PPF) scaffold. The biomaterial was able to release 80% of PTH in 3 days. Usually, growth factors have a short half-life,^165^ but the bioactivity of the biomolecule was confirmed and lasted until day 21. This biomaterial was tested in critical-sized femoral defects of Male Sprague–Dawley rats. There was no complete bone union; however, all samples in the 10 μg PTH condition evidenced ECO because the two sides of the defect were connected by bone or a mixture of bone and hypertrophic chondrocytes.^164^ These are promising results compared with the other conditions and nonloaded biomaterials conditions. Moreover, a controlled delivery system of BMP-2 and IL-8 was created using mesoporous bioactive glass coated with PEG (MBG/PEG) to promote ECO, particularly to improve stem cell recruitment and the creation of cartilage templates.^166^ The authors intended a rapid release of IL-8 obtained through its absorption into a polymer without crosslinking bounds and a slower and prolonged release of BMP-2, obtained by trapping this molecule in mesopores with similar diameters. Initially, the controlled delivery of IL-8 and BMP-2 was studied *in vitro* by analyzing the profile release of both molecules. Then, the BMP-2 and IL-8 loaded scaffold was implanted for 12 weeks in radius large defects of New Zealand white rabbits. IL-8 showed to increase the recruitment of stem cells compared with BMP-2. In addition, IL-8 increased the expression of chondrogenic genes, which enhanced the formation of large cartilage templates and the expression of BMP receptors. Consequently, with the release of BMP-2 and a higher number of receptors of BMP molecules, the transformation of the templates into bone tissue was significantly boosted.^166^ Generally, one of the disadvantages of using growth factors loaded in scaffolds to treat critical-sized defects is due to the supraphysiological quantity required, which may cause severe side effects.^167^ Thus, various cell-free approaches do not use growth factors in their formulation because of their short half-life time, the increased costs, and possible immunogenicity and toxicity.^165^^,^^168^ So, the next generation of approaches has provided other mechanical and structural cues to fulfill the lack of biochemical supplementation. For instance, the physiochemical properties of scaffolds can guide the biological processes toward endochondral regeneration. Scaffold porosity became increasingly relevant in bone repair and has been the subject of several studies. 3D printing β-tricalcium phosphate scaffolds with three different porous sizes, namely 100, 250, and 400 μm, were implanted to improve bone repair (Figure 3D).^147^ Calcium phosphate materials are widely applied in bone TE strategies.^169^, ^170^, ^171^ The scaffolds were placed in tibia bone defects, a long bone model of New Zealand rabbits inherently associated with ECO repair. Animals were euthanized at weeks 1 and 2 and weeks 4 and 8 for vascularization and bone repair evaluation, respectively. During the two first time points, cells were collected from the scaffold, and their protein expression was analyzed by western blot (Sox9, Col II, Runx2, Col I, and VEGF). The 400 μm scaffold showed an increased expression of Sox9 and Col II in the first week and higher vascularization in the second week, assessed by VEGF staining and immunochemistry assay, than the other two scaffolds. At week 4, histological sections of the scaffold were used for H&E and Masson’s trichrome staining, and the 400 μm scaffold presented more mineralized bone tissue, whereas the other conditions presented enhanced connective tissue. At the end of the experiment (week 8), the 400 μm scaffold achieved the most significant bone repair outcome.^147^ Beyond the importance of porosity of the scaffold, the pore alignment was also relevant in the induction of ECO. To prove it, three different collagen scaffolds, with a pore network alignment perpendicular to bone marrow or random alignment, were tested *in vitro* with seeded BMMSCs and then, *in vivo*, with cell-free implantation in femoral bone defects of Sprague-Dawley rats.^172^ The collagen scaffold with the same pore alignment as the bone marrow allowed to obtain ECO repair across the bone defect and higher host cell recruitment. Although tissue vascularization proved easier in the other scaffolds, the present vascularization was enough to improve tissue repair.^172^ The photofunctionalization of biomaterials has been also employed to enhance the potential of bone regeneration. A biomaterial with a dome-like structure composed of cobalt-chromium-molybdenum alloy was tested with and without UV-C irradiation when implanted in rabbit tibiae. The goal was to understand if the photofunctionalization enhances bone formation. This approach showed bone formation by ECO; however, more conclusive results are needed.^173^

As mentioned earlier, hypoxia is described as a favorable condition to enhance ECO bone formation. Thus, a biomaterial able to generate hypoxia conditions was applied *in vitro* and *in vivo* to recapitulate the ECO process. This biomaterial consists of an injectable hydrogel constituted by poly(glycerol sebacate)-co-poly (ethylene glycol)/polyacrylic acid (PEGS/PAA) that induces hypoxia by iron chelation. The experiment *in vitro* used macrophages-like cells and HUVECs to understand if the hydrogels with BMMSCs could polarize the macrophages and induce angiogenesis, respectively. Furthermore, the expression of the hypoxia-inducible factor 1α by macrophage-like cells was evaluated because it induces early-stage chondrogenesis and late-stage angiogenesis in ECO process repair. The hypoxia condition caused the HIF-1α expression and, consequently, bone repair. A stable HIF-1α expression was essential to obtain higher efficiency ECO bone repair.^163^

During the bone repair process, both ECO and IMO can simultaneously occur.^174^ Inspired by this concept, four nanocrystalline hydroxyapatite (nHA)−poly(thioketal urethane) cement were tested *in vivo* for bone repair in femoral defects of New Zealand white rabbits. The ossification and integration of the cement were analyzed for 18 months. Four types of cement were tested, an injectable cement and three putties, prepared by adding calcium phosphate, sucrose, or a combination of both. Calcium phosphate cement was utilized as a control. A combination of IMO and ECO was visible during cement integration; however, it was possible to verify the occurrence of ECO repair inside the created cement, whereas the controls only presented cement replacement by bone in the periphery.^175^ The most promising cell-free strategies are summarized in Table 3.

**Table 3:** ECO cell-free approaches

Authors/Reference	Biomaterial	Previous treatment/stimuli	*In vivo* study	Outcome
Wojda et al.^164^	Poly-propylene fumarate (PPF)	Parathyroid-hormone-loaded biomaterial	12 weeks	80% of PTH was released in the first 3 days and the remaining amount was until day 14. The developed biomaterial was tested in critical-sized femoral defects in rats. There was no complete bone union; however, all samples in the 10 μg PTH condition evidenced ECO because both sides of the defect were connected by bone or a mixture of bone and hypertrophic chondrocytes.
Diao et al.^147^	β-Tricalcium phosphate	Three different porous sizes: 100, 250, and 400 μm	8 weeks	Vascularization and bone repair evaluation were assessed for up to 8 weeks. The 400 μm biomaterial showed an increased expression of chondrogenic markers and higher vascularization in the second week compared with the other two biomaterials. At week 4, the 400 μm biomaterial presented more mineralized bone tissue, whereas the other conditions presented more connective tissue. This biomaterial achieved the most significant bone repair outcome at the end of the experiment.
Petersen et al.^172^	Collagen	Three different porous alignments: Equal to the bone marrow, perpendicular to bone marrow, or random alignment	6 weeks	The collagen scaffold with the same pore alignment as the bone marrow allowed to obtain ECO repair across the bone defect and higher host cell recruitment. Although tissue vascularization proved easier in the other scaffolds, the present vascularization was enough to improve tissue repair.
Zuchuat et al.^173^	Cobalt–chromium–molybdenum alloy	Photofunctionalization with UV-C irradiation	6 weeks	The results obtained by X-ray, micro-CT analysis, and H&E histology showed evidence of bone formation by ECO.
Lin et al.^166^	Mesoporous bioactive glass/poly-(ethylene glycol) (MBG/PEG)	IL-8 and BMP-2 loaded biomaterial	12 weeks	The release of IL-8 enhanced stem cell recruitment and the expression of chondrogenic genes. Consequently, the formation of large cartilage templates and the expression of BMP receptors were enhanced. BMP-2 release resulted in an accelerated formation of bone tissue. The developed system obtained the highest bone repair results, presenting increased tissue mineralization.
McGough et al.^175^	Nanocrystalline hydroxyapatite–poly(thioketal urethane) nanocomposites	Formulations: injectable, flowable cement and three moldable putties with varying ratios of calcium phosphate to sucrose granules	4, 12, and 18 months	Four nanocrystalline hydroxyapatite (nHA)−poly(thioketal urethane) cement were tested *in vivo.* The ossification and integration of the cement were analyzed for 18 months. A combination of IMO and ECO was visible during cement integration. It was possible to verify the occurrence of ECO repair inside the created cement.
Liu et al.^176^	Hierarchical macro/micro/nanoporous mesoporous bioglass (MBG) scaffold	DEX and rhBMP were loaded to the scaffold	4 weeks	DEX and rhBMP were loaded in a porous mesoporous bioglass scaffolds. The proposed scaffold stimulated the rapid chondrogenic differentiation by activating Hif-3α signaling pathway of MSCs.

### Conclusions and future perspectives

The IMO process inspires most bone TE strategies. With the recent developments of bone TE, ECO has been considered a key tool to solve the main drawback of the preview strategies, namely the lack of vascularization of the mineralized microtissues. The existence of an intermediate hypertrophic cartilage template allows the production of not only osteogenic but also angiogenic factors that induce bone formation/repair while recruiting the host blood vessels. ECO approaches reported in the literature are very diversified, using different cell types, biomaterials, culture media, and stimuli timings. The basic concepts of these engineered strategies are now becoming clear. Regarding the types of cells employed, MSCs and ECs co-culture is the most promising setup due to the described crosstalk between them, with the hypertrophic cartilage template in the native environment of a bone fracture. Thus, the combination of such co-culture with hypertrophic chondrocytes can promote the osteogenic differentiation of the MSCs and the recruitment of ECs. However, optimal co-culture conditions are not yet well established. The process of cell culture usually comprises two main steps. Firstly, the cells are cultured with chondrogenic supplementation factors to create the cartilage template. Subsequently, cells are cultured with hypertrophic supplementation factors, which are quite similar to osteogenic factors. The hypertrophic medium is essentially an osteogenic medium but applied to cartilaginous tissue, which results in a different output.

Besides the well-known scaffold-based approaches, there are also scaffold-free and cell-free approaches. Both approaches present examples where ECO repair is achieved. Of note, different scaffold-free approaches have been shown to enhance the ECO process, even without hypertrophic supplementation, by using, for example, pulsed electromagnetic field stimuli.^134^ In addition, iPSCs pellets previously differentiated into hypertrophic chondrocytes *in vitro* successfully created well-vascularized bone tissues after implantation and without teratoma creation. Therefore, banking multiethnic human leukocyte antigens (HLA)-homozygous iPSCs, and genome-editing strategies to engineer HLA matching in allogeneic settings via CRISPR-Cas9, can potentially replace the use of autologous cells.^177^, ^178^, ^179^ Cell-free and growth-factor-free approaches have also been demonstrated to enhance ECO repair, which could be achieved through the optimization of the network porosity or degradation rate of the scaffolds. In fact, the most important features of biomaterials for ECO approaches are the following: the diffusion of the nutrients; composition and stiffness, which should resemble the native ECM; and the porosity (∼100–400 μm) that allows the scaffold invasion by blood vessels when implanted. Studying these physicochemical properties of scaffolds will enable the development of a strategy that guides the biological processes toward endochondral regeneration. Regarding biomaterials type, collagen is the most used polymer for ECO repair, although other natural and synthetic polymers are also being investigated. On the other hand, the “minimalist-engineering” concept should also be considered when designing new strategies. Reducing the amount of scaffolds while guiding the performance and recruitment of a patient’s cells to the injury site to balance the regenerative niche toward the healing process should be the next step for better TERM approaches.^155^

Overall, the generation of *in vitro* ECO models remains reduced because most studies comprise the *in vivo* step for the hypertrophic state of previously primed cartilaginous templates. We believe that *in vitro* approaches can also be helpful for a better insight into the complete understanding and characterization of the ECO process. Furthermore, repairing critical-sized defects in human patients is challenging and presents several limitations, including time consumption and high costs. For instance, these engineered approaches need to fit the defect. Depending on the methodology, it may require a large amount of cells or the release of growth factors at supraphysiological levels to treat critical-sized defects.^167^ Thus, the proposed strategies should satisfy the mechanical properties and biocompatibility necessary for bone repair while fulfilling commercial requirements for translation into clinical practice. Even so, the repair of bone through ECO approaches holds tremendous power for clinical translation and the bone TERM field overall.
